# Controlled synthesis and size effects of multifunctional mesoporous silica nanosystem for precise cancer therapy

**DOI:** 10.1080/10717544.2018.1425779

**Published:** 2018-01-15

**Authors:** Bin Ma, Lizhen He, Yuanyuan You, Jianbin Mo, Tianfeng Chen

**Affiliations:** The First Affiliated Hospital, and Department of Chemistry, Jinan University, Guangzhou, PR China

**Keywords:** Size effect, anticancer, multidrug resistance, mesoporous silica nanosystem

## Abstract

Nanomaterials-based drug delivery systems display potent applications in cancer therapy, owing to the enhanced permeability and retention effect and diversified chemical modification. In this study, we have tailored and synthesized different sized mesoporous silica nanoparticles (MSNs) through reactant control to investigate the relevancy of nanoparticle size toward anticancer efficacy and suppressing cancer multidrug resistance. The different sized MSNs loaded with anticancer ruthenium complex (RuPOP) and conjugated with folate acid (FA) to enhance the selectivity between cancer and normal cells. The nanosystem (Ru@MSNs) can specifically recognize HepG2 hepatocellular carcinoma cells, thus enhance accumulation and selective cellular uptake. The smaller sized (20 nm) Ru@MSNs exhibit higher anticancer activity against HepG2 cells, while the larger sized (80 nm) Ru@MSNs exhibit higher inhibitory effect against DOX-resistant hepatocellular carcinoma cells (R-HepG2). Moreover, Ru@MSNs induced ROS overproduction in cancer cells, leading to DNA damage and p53 phosphorylation, consequently promoting cancer cells apoptosis. Ru@MSNs (80 nm) also inhibited ABCB1 and ABCG2 expression in R-HepG2 cells to prevent drug efflux, thus overcome multidrug resistance. Ru@MSNs also inhibited tumor growth *in vivo* without obvious toxicity in major organs of tumor-bearing nude mice. Taken together, these results verify the size effects of MSNs nanosystem for precise cancer therapy.

## 1. Introduction

Nanotechnology-based delivery systems display potent applications in cancer therapy, owing to the enhanced permeability and retention (EPR) effect of nanoparticles (Talelli et al., 2015; Yu & Zheng, 2015). Nanoparticles with EPR effect permeate into tumor sites from its leaky epithelium and discontinuous microvasculatures, which formed by the rapid growth of tumors (Peer et al., 2007). Recent studies have supported that particle size; morphology and surface property are the important factors impact their EPR effect (Matsumura & Maeda, 1986). Nanometer level particle size plays an essential role in tumor cellular accumulation, uptake, and retention (Cheng et al., 2012; Pan et al., 2012). For example Tan et al. reported that 48 nm multifunctional PEG-MSNPs-CD-PEG-FA more easily accumulated in tumor than same nanosystem at 100 nm in MDA-MB-231 tumor-bearing mice (Zhang et al., 2014). Zhou et al. synthesized a polymer micelles PELEss-DA (46 nm) could accumulate in tumor under acidic condition and were altered to 32 nm by GSH improving nuclear accumulation (Guo et al., 2015). Langer et al. found that although nanoparticles less than 400 nm can extravasate from leaky vasculature into tumor microenvironments, only 10–100 nm particles avoid liver capture and renal filtration, enhancing efficient cellular uptake (Peer et al., 2007; Danhier et al., 2010). Furthermore, Liang et al. reported smaller particles achieve better tumor microenvironment penetration (Huo et al., 2013), and Kjems et al. found small particles more easily return to blood circulation leading to lower retention efficiency (Larsen et al., 2009). Therefore, appropriate particle size is an essential factor to achieve enhanced cellular uptake and tumor retention. Nanocarriers offer unique superiorities for cancer targeted drug delivery, including higher efficiency and lower toxicity compared with conventional chemotherapeutics, which show great potential for cancer chemotherapy. Many different nanomaterials have been reported as drug carriers for cancer therapy, such as selenium nanoparticles, liposomal, oxides, proteins, and polymers (Shen et al., 2014; Huang et al., 2016; Song et al., 2016; Shi et al., 2017). Among these nanomaterials, mesoporous silica nanoparticle (MSN) provide superior drug delivery system options because of high drug loading capability, high biocompatibility, low toxicity, especially the controllable particle size and pore size (He et al., 2014a; Jiang et al., 2014; Hu et al., 2015; Chen & Shi, 2016). Besides, MSNs are popular for targeted cancer treatments due to their easy surface modification (You et al., 2015; Liu et al., 2016). Surface ligands like antibodies, polypeptide and folate acid (FA) that can preferentially recognize tumor cell surface biomarkers and enhance selectivity between tumor and normal cells, is of great significance (Lu & Low, 2002; Luo et al., 2013; He et al., 2015). Folate receptor (FR) in cancer cells membrane is overexpressed by comparing with corresponding normal cells (Devanand Venkatasubbu et al., 2013; Hansen et al., 2015). These kinds of epigenetic differences between tumor and normal cells make FA regarded as a useful ligand for specific recognition of cancer cells, thus provide a good strategy for rational design of cancer-targeted nanomedicine for cancer therapy.

Cancer is one of the most serious threats toward human health worldwide, and it provides many extremely difficult challenges in the fields of chemistry, pharmacy, bioscience, and clinical medicine (Torre et al., 2015). Conventional chemotherapeutic drugs, such as cisplatin, oxaliplatin, and paclitaxel are undisputed therapeutic agents for clinical practice, but the drawback of them including tissue toxicity, poor tumor selectivity, and multidrug resistance (MDR) impede their broader applications (Blanco et al., 2015). Importantly, MDR leads to drug efflux in tumors causing inefficient treatment and significantly hampering expansion of cancer chemotherapy. Overcoming MDR has been an important focus for improving cancer chemotherapy efficacy and preventing tumor recurrence. Studies have found that one of the mechanisms of MDR is the overexpression of ATP-binding cassette (ABC) superfamily of transporters in cancer cells, such as P-glycoprotein (P-gp/ABCB1), MDR-associated protein 2 (MRP2/ABCC2), and breast cancer resistance protein (BCRP/ABCG2) (Wu et al., 2014; Kathawala et al., 2015; Song et al., 2016). Therefore, inhibiting the expression of these efflux transporters has become one of the important approaches to suppress cancer MDR (Holohan et al., 2013; Wang et al., 2015; Wu et al., 2016). For example Zink et al. synthesized MSNs co-delivery system of DOX and P-gp siRNA to overcome multidrug resistant and enhance cancer therapy in MCF-7/ADR cells via knocking down of *P-gp* gene by siRNA (Meng et al., 2013). Zou et al. utilized single-walled carbon nanotubes (SWNTs) conjugated with anti-P-gp antibody to anchor the overexpressed P-gp on human leukemia cells of K562R and suppress the proliferation of multidrug-resistant cells (Li et al., 2010). Furthermore, Shi et al. also found that TAT-peptide modified MSNs loading DOX could promote nanoparticles across nuclear membrane and drug release in nucleoplasm avoiding drug efflux via P-gp thus overcome MDR (Pan et al., 2013; Chen et al., 2014). According to our previous report, we tailored the particle size of DOX@MSNs nanosystem and optimized the particle size of nanosystem that could effectively penetrate BBB and targeted the tumor tissue to achieve enhanced anti-glioma efficacy (Mo et al., 2016). However, the size effects on liver cancer treatment and the action mechanisms remain elusive.

**Scheme 1.**
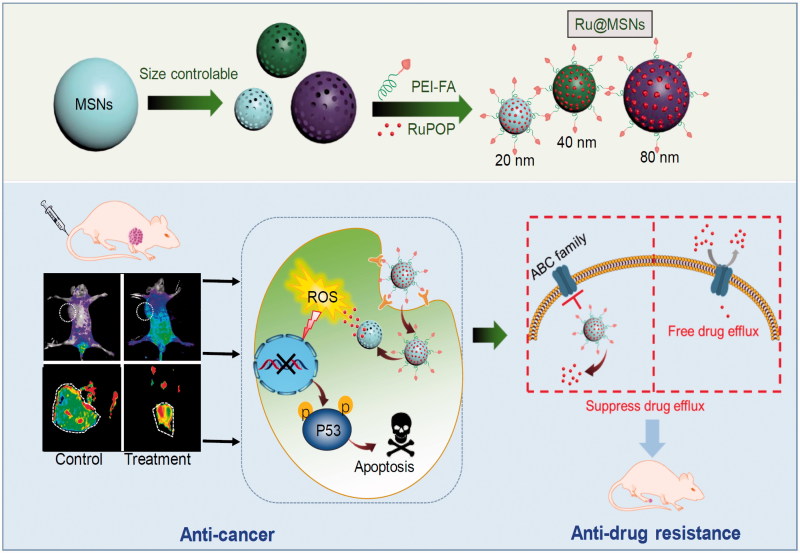
Rational design and synthesis of different-sized MSN nanosystems to enhance the anticancer activity and suppress cancer multidrug resistance.

In this study, size-dependent MSN nanoparticle has been tailored, loaded with ruthenium complex (RuPOP) and modified with cancer targeted PEI-FA polymer to enhance anticancer effects and illuminate size effect in cancer therapy (Scheme 1). Coating of FA-conjugated polyethyleneimine (PEI-FA) on MSN surfaces can block the nanoparticle’s nanochannels, preventing the loaded drug from pre-releasing in blood circulation. PEI modification also changes the zeta potential from negative to positive, enhancing the stability and internalization in tumor cells due to the negatively charged of cell membrane (He & Shi, 2014). Importantly, we have investigated the relevancy of nanoparticle size toward cellular uptake and tumor retention, and finally, influence the anticancer efficacy and suppressing cancer MDR.

## 2. Materials and methods

### 2.1. Materials

Diethanolamine (DEA), triethanolamine (TEA), cetyltrimethylammonium chloride (CTAC), cetyltrimethylammonium bromide (CTAB), tetraethyl orthosilicate (TEOS), 1-(3-(dimethylamino)-propyl)-3-ethylcarbodiimide hydrochloride (EDC), N-Hydroxysuccinimide (NHS), poly (etherimide) (PEI, Mw =10,000), and folic acid (FA) were purchased from Aladdin Chemistry Co., Ltd. (Shanghai, China). Ruthenium complex (RuPOP) was synthesized according to previous work (Chen et al., 2010). The drug concentration in all biological studies was calculated as RuPOP by ICP-MS analysis.

### 2.2. Cell lines

Hepatocellular carcinoma HepG2 cells, human normal liver L02 cells, and DOX-resistance R-HepG2 cells were purchased from American Type Culture Collection (ATCC, Manassas, VA). HepG2 and L02 cells were incubated in DMEM medium supplemented with fetal bovine serum (10%), 100 units/mL of penicillin, and 50 units/mL of streptomycin at 37 °C in 5% CO_2_ incubator under 95% relative humidity. R-HepG2 cells were incubated in DMED medium containing DOX (800 ng/mL) in the same condition.

### 2.3. Synthesis of different-sized Ru@MSNs nanosystems

The synthesis of different-sized of MSNs was based on our previous reports (Mo et al., 2016). Three different-sized of MSNs (20, 40, and 80 nm) were chosen to deliver RuPOP to tumor and functionalized with PEI-FA as a target agent. In detail, 20 mg of RuPOP was dissolved in 10 mL DMSO. Then 50 mg of different-sized MSNs were suspended into the solution and stirred for 24 h at room temperature, respectively. The nanoparticles were obtained by centrifugation at 12,000 rpm for 10 min and mixed with pre-prepared PEI-FA solution for 24 h. Finally, different-sized Ru@MSNs was obtained by centrifugation and low-temperature vacuum drying.

### 2.4. Characterization of different-sized Ru@MSNs nanosystems

Transmission electron microscopy (TEM, Hitachi (H-7650), 80 kV, Tokyo, Japan), N_2_ adsorption-desorption isotherm (NOVA 4200e surface area analyzer (Quantachrome)), Nano-ZS instrument (Malvern Instruments Limited, Malvin City, England), Fourier transform infrared spectroscopy (FTIR, Equinox 55, Bruker, Bly Raica, Massachusetts, USA) spectrometer, UV–Vis–NIR absorption spectra (UH-4150 Spectrophotometer, Hitachi, Tokyo, Japan), and fluorescence spectrometer (Thermo Fisher Scientific, Massachusetts, USA) were used to determine the morphology and structure of different-sized MSNs.

### 2.5. MTT assay

The cells at a density of 2 × 10^4^ cells/mL were pre-seeded in 96-wells plate (0.1 mL/well) for 24 h, and then treated with 20, 40, and 80 nm Ru@MSNs at different concentrations. After treated for 72 h, 5 mg/mL MTT was added into the well (25 μL/well) and incubated at 37 °C for 3 h. And then the medium was removed. The precipitate was dissolved by DMSO. The absorbance was detected by microplate spectrophotometer (SpectrAmax 250, Marshall Scientific, Hampton, NH) with the wavelength at 570 nm.

### 2.6. Cellular uptake of Ru@MSNs

Of, 8 × 10^4^ cells/mL of HepG2, R-HepG2, and L02 cells were pre-seeded in 96-wells plate (0.1 mL/well) for 24 h to quantify cellular uptake of different-sized Ru@MSNs. Nanosystems were added into the wells at the final concentration at 1 μM and the cells were incubated for 4 and 8 h, respectively. After that, the medium was removed and each well was washed with pre-cold PBS for three times to remove extra nanoparticles. The cells were lysed with 100 μL of Tritonx-100. Finally, the fluorescence intensity of RuPOP was detected by the fluorescence microplate reader (SpectraMax M5, MD) and standard curve, with the excitation and emission wavelengths set at 479 and 599 nm, respectively.

### 2.7. FA-competing assay

The cells were pretreated with excess amount of FA (1 mg/mL) followed by the incubation of Ru@MSNs. The cellular uptake efficacy of Ru@MSNs in cells was determined as mentioned above.

### 2.8. Drug retention efficacy of Ru@MSNs

Of, 20 × 10^4^ cells/mL of HepG2 and R-HepG2 were pre-seeded in 2 cm culture dish for 24 h to analyze the drug retention effect of different-sized Ru@MSNs. After attachment, the cells were pretreated with 2 μM of Ru@MSNs for 6 h and the medium was removed. Each dish was washed with PBS for three times to remove extra nanoparticles. Of, 2 mL of fresh medium was added. Finally, the fluorescence intensity of RuPOP the in supernatant was determined within 24 h.

### 2.9. *In vitro* drug release of Ru@MSNs

Of, 1 mg/mL of different-sized Ru@MSNs in PBS (pH = 7.4) and HepG2 cells lysate was moderately stirred at 37 °C in glass reactor, respectively. Of, 100 μL of supernatant was taken out at specific times to determine the concentration of RuPOP by the fluorescence microplate reader with the excitation and emission wavelength at 479 and 599 nm, respectively. Then, the fresh PBS and cell lysate were added into the solution to keep the volume invariability.

### 2.10. Real-time living cell monitoring

6 × 10^4^ cells/mL of HepG2 cells were seeded in 2 cm culture dishes for 24 h, and then treated with 0.1 μM of different-sized Ru@MSNs to detect the cellular location of nanosystems via fluorescence microscope (EVOS FL, Beijing, China) at specific times. Cell nucleus was stained by 1 *μg*/mL of Hoechst 33342 for 20 min.

### 2.11. Hemolysis analysis

The human blood, which comes from the first affiliated hospital of Jinan University, was centrifuged at 3000 rpm centrifuge for 10 min to separate the red blood cells. The red blood cells solution treated with 0.4 mL Ru@MSNs (1 μM) were incubated at 37 °C for 10 min and 3 h. Meanwhile, the PBS was used as a positive control and the Tritonx-100 was served as negative control. At the specific times, the red blood cells solution was centrifuged at 3000-rpm centrifuge for 10 min and 100 μL of supernatant was taken out to calculate the hemolysis rate at 540 nm using a microplate spectro-photometer (SpectrAmax 250, Marshall Scientific, Hampton, NH).

### 2.12. Flow cytometric analysis

Flow cytometric analysis was employed to determine the cell cycle distribution of HepG2 cells after drug treatments. Briefly, 2 × 10^4^ cells/mL of HepG2 and R-HepG2 cells were seeded in 6 cm culture dish for 24 h, and incubated with 0.2 μM different-sized Ru@MSNs for 24 h. And then, the cells were digested and fixed with 70% ethanol for 12 h at −20 °C. The fixed cells were stained with PI solution for 0.5 h and analyzed using Epics XL-MCL flow cytometer (Beckman Coulter, Miami, FL). Then Multi Cycle software (Phoenix Flow Systems, San Diego, CA) was used to calculate the cell cycle distribution of the cells.

### 2.13. ROS generation

2 × 10^5^ cells/mL of HepG2 and R-HepG2 cells were pre-seeded in 96-wells plate (0.1 mL/well) for 24 h, then the cells were incubated with DHE (10 μM) at 37 °C for 30 min. After extra DHE removed, 1 μM of different-sized Ru@MSNs were added into the cells, and then the fluorescence intensity of DHE was measured by a fluorescence microplate reader (SpectraMax M5, MD) to evaluate the ROS variation.

### 2.14. Immunofluorescence analysis

The cells at a density of 20 × 10^5^ cells/mL were pre-seeded in 2 cm culture dish and cultivated for 24 h. The cells were exposed to Ru@MSNs (1 μM) for 6 h and then the immunofluorescence analysis was used to examine the expression level of phosphorylational p53.

### 2.15. Western blot analysis

The cells (1 × 10^5^ cell/mL) were pre-seeded in 10-cm culture dish for 24 h and treated with different-sized Ru@MSNs (1 μM) for 24 h. The total cellular proteins were extracted by cell lysis buffer and protein concentrations were examined by BCA assay. The effects of Ru@MSNs on the expression levels of proteins were determined by Western blotting. The expression of β-actin was used as internal standard to analyze the amount of protein in each lane.

### 2.16. *In vivo* fluorescence imaging

All nude mice experiments were approved by the Institutional Animal Care and Use Committee of Jinan University. HepG2 cells (2 × 10^6^) were injected subcutaneously into the nude mice ages 6 weeks old. When the volume of tumors was growth into 200 mm^3^, the mice was treated with different drugs (0.2 mg/kg) via tail intravenous injection. The biodistribution of RuPOP and different-sized Ru@MSNs was detected at 12, 24, 48, and 72 h post-injection by using life imaging system (Xenogen, Caliper Life Sciences, Hopkinton, MA). Meanwhile, at different times, three mice in every group were killed and the normal organs and tumor were obtained to perform further fluorescence imaging observation.

### 2.17. *In vivo* antitumor experiment

HepG2 tumor-bearing nude mice were divided into five groups (*n* = 8 for each group). When the volume of tumors was growth into 200 mm^3^, the mice was treated with different drugs (0.2 mg/kg) via tail intravenous injection. The body weight and perpendicular diameter of tumor were detected every day to calculate the tumor volume based on the formula:

$$Tumor\;volume\;({mm}^{3})=\;\frac{1}{2}\times length\times {width}^{2}.$$

All nude mice were dissected after 3-weeks treatment. Blood samples were collected for hematology analysis. Organs including heart, spleen, lung, liver, kidney, and tumor were collected for H&E staining.

### 2.18. Statistics analysis

All the data were expressed as mean ± standard deviation. Differences between the control and experimental groups were analyzed by a two-tailed Student’s *t* test. One-way analysis of variance was used in multiple-group comparisons, and statistical analysis was performed by using SPSS statistical program version 13 (SPSS Inc., Chicago, IL). Significant differences between the control and treatment groups are indicated at *p < .05.

## 3. Results

### 3.1. Size-controlled synthesis and characterization of Ru@MSNs nanosystems

Nanoparticle size shows significant impact on drug delivery, cellular uptake, and anticancer effect (He et al., 2014b). Therefore, we tailored different-sized MSNs and then investigated the relationship between particle size and anticancer activity. First, we designed and synthesized a variety of different-sized MSNs using reactant type and ratio according to previous reports. Base catalyst and template had great influence in controlling the particle size of MSNs (Wu et al., 2013; Mo et al., 2016). For example under the same condition with 0.3 vt% TEA as base catalyst and 0.75 vt% TEOS as silica source, MSNs at 60 or 80 nm was obtained with 10 wt% CTAB or CTAC as templates, respectively. These results suggested that different templates influence the particle sizes to some extent. In addition, different base catalysts were also essential in size regulating. For example the concentration of DEA was involved in regulating MSNs particle size. The same concentration of silica source (TEOS) and template (CTAB), but different DEA (0.2 and 0.6 vt%) created 20 and 40 nm MSNs, respectively. Therefore, through controlling the templates and base catalysts, different-sized MSNs were successfully synthesized.

**Figure 1.**
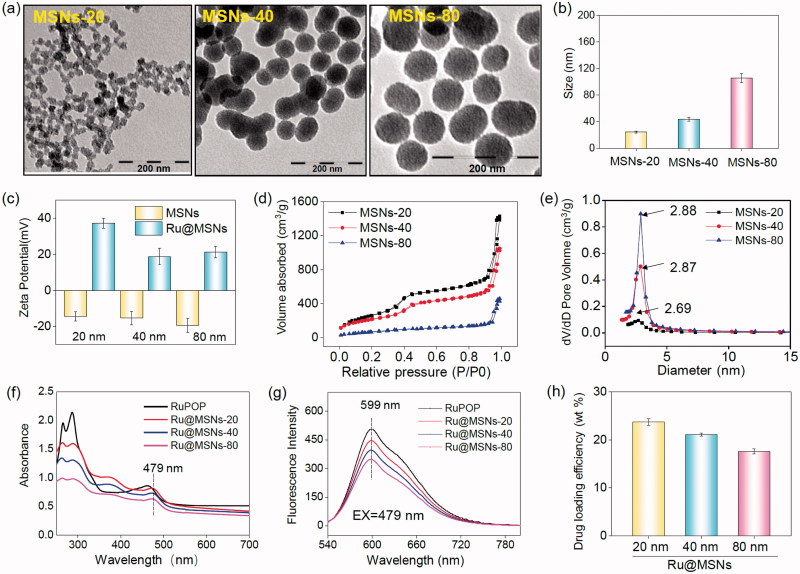
Morphology and structural characterization of different-sized MSNs. (a) TEM images of the different-sized MSNs (scale =200 nm). (b) Sizes distribution of the different-sized MSNs. (c) Zeta potential for three different-sized MSNs and Ru@MSNs nanosystems. (d) Nitrogen adsorption and desorption isotherms for the different-sized MSNs. (e) Pore sizes distribution for the different-sized MSNs. (f) UV and (g) fluorescence spectra of RuPOP and Ru@MSNs. (h) RuPOP loading efficiency in the three different-sized MSNs nanosystems. Values expressed were mean ± standard deviation of triplicates. Value represents means ± SD (*n* = 3).

Therefore, these different-sized MSNs nanoparticles were chosen to carry RuPOP and functionalize with PEI-FA to synthesize different-sized Ru@MSNs as cancer-targeted nanosystems. Microscopic and spectroscopic analyses were utilized to characterize the MSNs and Ru@MSNs and examine their properties. As shown in Figure 1(a), the TEM images showed that the mean diameters of the three different-sized MSNs particles were about 20, 40, and 80 nm, and all possessed excellent monodispersity and uniformity. Zetasizer Nano-ZS average diameter analysis of these MSNs (Figure 1(b)) exhibited a little larger than TEM measurements (about 24.4, 43.8, and 105.6 nm), which were influenced by hydrodynamic ratios of this analyzer. Therefore, the above results proved that three different-sized MSNs were controllable synthesis. After MSNs loaded with RuPOP and modified by FA-PEI, zeta potentials of 20, 40, and 80 nm Ru@MSNs changed to positive charge from the electronegative MSNs. As shown in Figure 1(c), the free MSNs were negatively charged (−14.3, −15.3, and −19.4 mV, respectively), and Ru@MSNs possessed positive charge (37.3, 18.8, and 21.2 mV), resulting from plenty of amino groups from strongly electropositive PEI. Thus, PEI-FA encapsulated the surface of MSNs via electrostatic interactions leading to positive charge of Ru@MSNs, subsequently the change of the zeta potentials could improve the stability of these nanosystems (Figure S1). In addition, as shown in Figure S2(a,b), the size (hydrodynamic diameter) of different-sized MSNs in FBS were 23.5, 44.6, and 86.3 nm, respectively, and the size in DMEM was 26.5, 48.6, and 105.9 nm. After 72 h, that size in FBS were 26.7, 45.8, and 95.6 nm and size in DMEM were 28.6, 50.5, and 114.7 nm. Although the hydrodynamic diameters of different-sized MSNs were slightly increased, the ‘protein corona’ may be formed on the surface of nanoparticles. But in general, the different sized-MSNs revealed good stability in fetal bovine serum and DMEM.

Furthermore, N_2_ absorption-desorption isotherm showed that the MSNs have a structure of cylindrical pores. As shown in Figure 1(d), three different-sized MSNs exhibited that adsorption branch of isotherm was inconsistent with the desorption branch conforming to type IV isotherm. In addition, the appearing of hysteresis loop indicated the structure of cylindrical pores of MSNs. The pore size of 20, 40, and 80 nm MSNs were about 2.88, 2.87, and 2.69 nm, respectively (Figure 1(e)).

The conjugation of FA to PEI was investigated by FTIR. As shown in Figure S3, the new peaks of 1631 and 1512 cm^−1^ could be assigned to amide bands I and II, respectively, which revealed that the amino group from PEI successfully bond to carboxyl donated from FA. The UV–Vis and fluorescence spectra were used to detect whether the optical properties of RuPOP were changed after loaded into MSNs pore. As shown in Figure 1(f), the absorption peak of RuPOP was 462 nm, while the absorption peak of different-sized Ru@MSNs was redshift to 479 nm. Consequently, under the excitation wavelength with 479 nm, the emission peak of Ru@MSNs was about 599 nm (Figure 1(g)). In a word, these results indicated that RuPOP was successfully loaded in the MSNs and maintained its properties. Different particle-sized MSNs showed different loading capacities of RuPOP. As shown in Figure 1(h), the drug loading efficiency of 20, 40, and 80 nm Ru@MSNs was about 23.7, 21.1, and 17.6%, respectively. The concentration of RuPOP loaded in different-sized MSNs was measured by ICP-MS. These results determined that small-sized MSNs exhibited better drug loading capacities than larger-sized nanoparticles.

### 3.2. Size effects on anticancer activity in vitro

**Figure 2.**
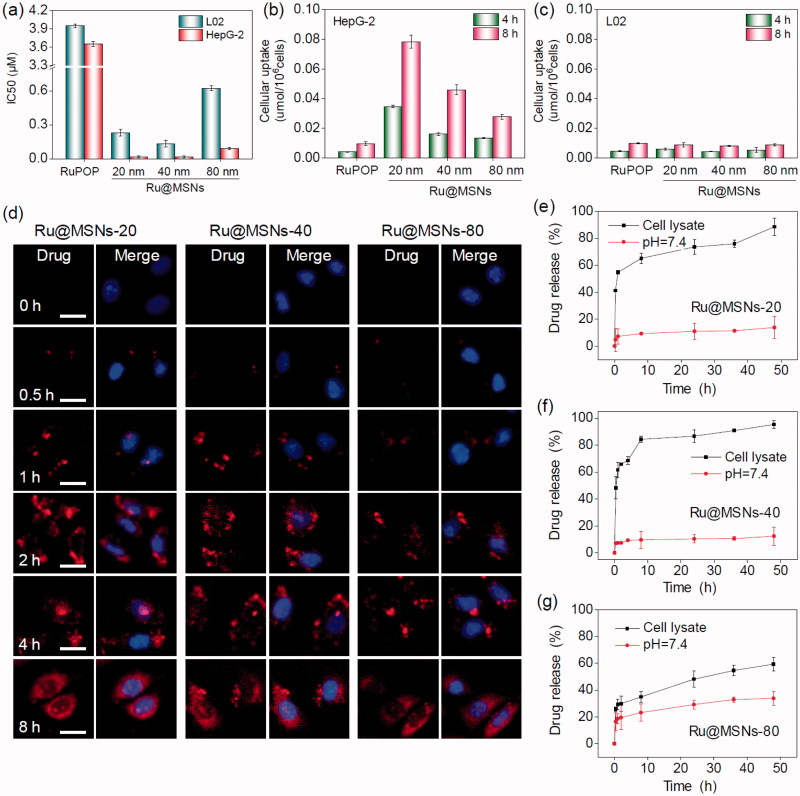
Anticancer activity and the selective cellular uptake of Ru@MSNs *in vitro*. The drug concentration in all biological studies was calculated as RuPOP by ICP-MS analysis. (a) IC_50_ for the RuPOP and different-sized Ru@MSNs toward HepG2 tumor cells and L02 normal cells. Cellular uptake of different-sized Ru@MSNs in HepG2 (b) and L02 (c) cells for 4 and 8 h. Cells were treated with 1 μM of different-sized Ru@MSNs and free RuPOP. (d) Localization of the different-sized Ru@MSNs (red) in HepG2 cells. The cells were treated with 1 μM of Ru@MSNs. (e), (f), and (g) *in vitro* drug release for the different-sized Ru@MSNs in PBS at pH =7.4 and cells lysate. The scale bar is 20 μm. Value represents means ± SD (*n* = 3).

Positive targeting is a critical strategy to improve the selectivity of nanoparticles between cancer and normal cells. In this study, we conjugated FA on the surface of different-sized Ru@MSNs and then examined their anticancer activity and toxicity in hepatocellular carcinoma cells (HepG2) and normal human liver cells (L02). The safety of MSNs carriers, anticancer efficacy, and cytotoxicity of Ru@MSNs was detected by MTT assay. As shown in Figure S4 the cells viabilities of different-sized MSNs carriers (20, 40, and 80 nm), MSNs-FA and PEI-FA with final concentration at 100 μg/mL which were far higher than cell experiments with Ru@MSNs *in vitro*, were 106.8, 98.3, 97.6, 94.5, 91.6, 95.5, and 93.6% in HepG2 cells, and 93.7, 95.1, 98.0, 96.8, 94.9, 93.4, and 92.6% in R-HepG2 cells, respectively, thus these results certified the nontoxicity of MSNs, MSNs-FA and PEI-FA *in vitro*. As shown in Figure 2(a), three Ru@MSNs nanosystems (20, 40, and 80 nm) all exhibited remarkable anticancer activities against HepG2 cells, which significantly higher than that of free RuPOP. For instance, the IC_50_ (as calculated by RuPOP) of 20 nm Ru@MSNs (0.017 μM) against HepG2 cells was reduced by approximately 215 times compared with free RuPOP (3.654 μM). Besides, the smaller particle sized Ru@MSNs showed higher therapeutic effects than the lager nanosystems. For example the IC_50_ of Ru@MSNs at 20, 40, and 80 nm against HepG2 cells was about 0.017, 0.018, and 0.091 μM, respectively. The safety index value (IC_50_ (L02)/IC_50_ (HepG2)) of the three Ru@MSNs showed significantly higher than that of free RuPOP (1.081), especially 20 nm of Ru@MSNs (13.529), subsequently confirming the nanosystems in this study had much lower cytotoxicity toward normal human liver cells than that of free RuPOP. These results suggested that Ru@MSNs possessed great selectivity between cancer cells and normal cells than the free RuPOP.

### 3.3. Size effects of nanoparticles on intracellular uptake

Cellular uptake is critical in achieving highly efficient anticancer activity and capability of decreasing toxic side effects. Therefore, we measured cellular uptake of different-sized Ru@MSNs in HepG2 and L02 cells. As shown in Figure 2(b,c), the uptake of Ru@MSNs in HepG2 cells were prominently higher than the free RuPOP after 4 and 8 h incubation. For instance, the drug concentration of Ru@MSNs at 20 nm in HepG2 cells were about 0.035 and 0.078 μmol/10^6^ cells after 4 and 8 h, respectively, while the intracellular drugs in the free RuPOP treatment group were about 0.004 and 0.009 μmol/10^6^ cells under the same conditions. In addition, L02 cells showed lower cellular uptake than HepG2 cells after treated with Ru@MSNs or free RuPOP. For example the drug concentrations of Ru@MSNs at 80 nm in L02 cells after 4 and 8 h treatment were only 0.005 and 0.009 μmol/10^6^ cells, respectively. These results indicated that the FA targeted Ru@MSNs showed excellent selectivity of cellular uptake between hepatocellular carcinoma and normal cells.

FR overexpression in hepatocellular carcinoma cell membranes is a key factor of high selectivity of Ru@MSNs. Therefore, we compared FR expression levels of L02, HepG2, and R-HepG2 cells using western blot analysis. As shown in Figure S5, the expression level of FR was higher in HepG2 and R-HepG2 cells than that of L02 cells. Consequently, the surfaces of Ru@MSNs were functionalized with FA which could preferentially bond to FR-overexpressed in HepG2 and R-HepG2 cell membranes, promoting sensitivity and selectivity between hepatocellular carcinoma and normal cells. FA-functionalized nanocarriers exhibited significantly superior selectivity toward FR-overexpressed cancer cells and were nontoxic to normal cells. To further confirm the FA receptor-mediated selective cellular uptake, we examined the intracellular drug concentration in HepG2 and L02 cells after pre-blocked by FA. As shown in Figure S6(a,b), after pretreatment with 1 mg/mL FA for 2 h, the drug concentrations of FA-functionalized Ru@MSNs were significantly inhibited in HepG2 and L02 cells. For example the drug concentration of Ru@MSNs at 20, 40, and 80 nm in FA pre-blocked HepG2 cells decreased to 0.0063, 0.0063, and 0.0059 μmol/10^6^ cells after 8 h treatment, and the inhibition of cellular uptake was about 92.7, 86.0, and 80.7%, respectively, compared with that without pretreatment cells. On the contrary, the cellular uptake in L02 cells displayed little inhibition after pretreated with FA. Therefore, FA modification could promote selective cellular uptake of Ru@MSNs in FR-overexpressed cancer cells. In addition, the intracellular retention of Ru@MSNs has great influence on the amount of drug in cancer cells and anticancer activity. Therefore, we examined the drug retention efficacy of the three different-sized Ru@MSNs nanosystems and free RuPOP in HepG2 cells. As shown in Figure S6(c), all the three particle sizes of Ru@MSNs nanosystems could remain for a longer time in HepG2 cells than the free RuPOP. Moreover, the smaller sized Ru@MSNs at 20 (94.2%) and 40 nm (91.7%) showed higher retention efficacy in HepG2 cells than that of the lager sized Ru@MSNs at 80 nm (80.7%) and free RuPOP (59.1%) even after 20 h. Taken together, compared with the free RuPOP, Ru@MSNs could accumulate in HepG2 cells more easily and remain for a longer time, thus revealing the higher anticancer effect in HepG2 cells.

### 3.4. Intracellular localization and translocation of Ru@MSNs

Endocytosis is a vital intracellular uptake mechanism of nanomaterials, such as MSNs. In this study, we utilized the strong red auto-fluorescence of RuPOP and Hoechst 33342 (blue) to investigate localization of the different-sized Ru@MSNs in HepG2 cells by fluorescence microscope. As shown in Figure 2(d), Ru@MSNs gathered in the cell membrane after 0.5 h incubation. And then the Ru@MSNs at 20 and 40 nm began to enter the cytoplasm after 1 h, which was faster than that of lager sized Ru@MSNs at 80 nm. After 2 h of incubation, Ru@MSNs at 20 and 40 nm were found a certain amount of accumulation in cytoplasm and the red fluorescence intensity of Ru@MSNs at 20 nm was stronger than Ru@MSNs at 40 nm, until this moment, Ru@MSNs at 80 nm began to enter cytoplasm. After 4 h of incubation, the accumulation of nanodrug in cytoplasm was increased and all three kinds of Ru@MSNs located in the cytoplasm at 8 h. These results demonstrated that the smaller sized Ru@MSNs (20 and 40 nm) permeated cell membranes easier than larger Ru@MSNs (80 nm), hence showed higher anticancer activity toward HepG2 cells.

### 3.5. *In vitro* drug release from the nanoparticles

Capable of controlling drug release, nanodrug delivery system could alleviate undesirable drug diffusion, thereby reducing toxic side effects toward normal tissues during blood circulation (Mekaru et al., 2015; Arosio & Casagrande, 2016). Therefore, we measured RuPOP released from the different sized Ru@MSNs in PBS at pH 7.4 and HepG2 cell lysate to simulate the environments of blood circulation and cancer cells, respectively. As shown in Figure 2(e–g), cumulative released of RuPOP in PBS at pH 7.4 after 48 h from Ru@MSNs at 20, 40, and 80 nm diameter were about 13.8, 12.5, and 33.9%, respectively. However, the different sized Ru@MSNs nanosystems had different drug release patterns in the intracellular environment of HepG2 cells. RuPOP released from Ru@MSNs at 20 and 40 nm ascended rapidly in HepG2 cell lysate, with cumulative drug release up to 65.1 and 84.3% after 8 h, then reaching to 88.6 and 95.4% after 48 h, respectively. While, the drug release pattern of Ru@MSNs at 80 nm was lower than the other two smaller sized nanosystems in HepG2 cell lysate, with the cumulative release about 48.5% after 48 h. These results demonstrated that all three-sized Ru@MSNs were stable during blood circulation, while under the intracellular conditions, RuPOP could release from the nanosystem in short order, especially for the nanosystem at 20 and 40 nm. Additionally, drug leakage from nanocarriers in blood circulation can trigger many biosecurity problems. Therefore, we examined the hemolysis rates for the different sized Ru@MSNs and free RuPOP. As shown in Figure S7, the hemolysis rates of Ru@MSNs and free RuPOP were all less than 5.0% after 3 h treatment, which confirmed the high hematological security for the nanodrug. Therefore, under intracellular condition, the different sized Ru@MSNs were capable to bio-responsively trigger RuPOP release from the carrier in a controllable pattern, and significantly enhance stability and hematological security in body blood circulation.

### 3.6. *In vitro* induction of cell apoptosis

**Figure 3.**
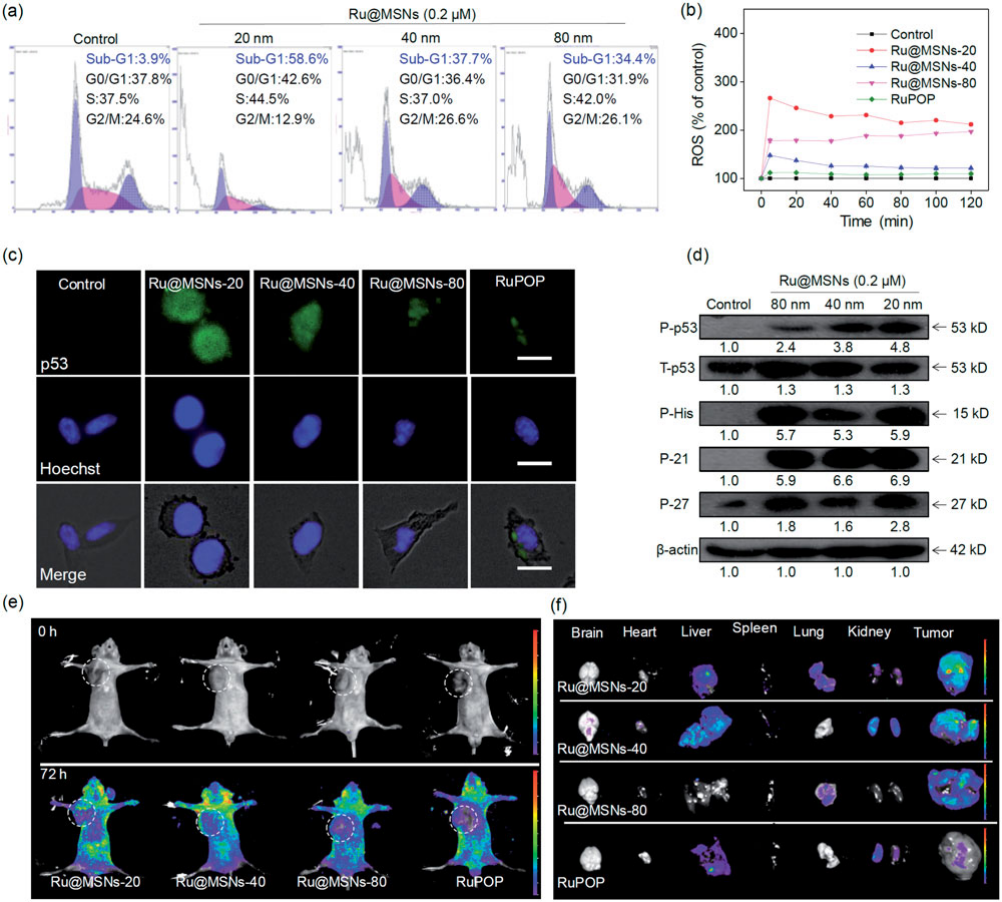
(a) Flow cytometric analysis of HepG2 cells (2 × 10^4^ cells/mL) exposed with 0.2 μM of different-sized Ru@MSNs for 24 h. (b) ROS overproduction in HepG2 cells (2 × 10^5^ cells/mL) exposed to RuPOP and Ru@MSNs (1 μM) for 2 h. (c) Immunofluorescence of phosphorylated p53 in HepG2 cells with different-sized Ru@MSNs and free RuPOP at 1 μM. The scale bar is 20 μm. Value represents means ± SD (*n* = 3). (d) Activation of p53 signal pathway in HepG2 cells exposed to different-sized Ru@MSNs. (e) Fluorescence imaging monitor the accumulation and distribution of the different-sized Ru@MSNs in HepG2 xenograft nude mice at 72 h. The treated concentration was 0.2 mg/kg of Ru@MSNs. (f) Fluorescence imaging of the main organs in 72 h-treatment with Ru@MSNs and free RuPOP.

Apoptosis and cell cycle arrest are two major action modes to causes cell growth inhibition and cell death (Morad & Cabot, 2013; Karimian et al., 2016). We used flow cytometry to detect HepG2 cell cycles after treatment and investigated anti-proliferation for the different sized Ru@MSNs nanosystems. We calculated the ratio of apoptotic cells from the sub-G1 peaks of hepatocellular carcinoma cells. As shown in Figure 3(a), all the three kinds of Ru@MSNs nanosystems enhanced HepG2 cell apoptosis, and exhibited different effects toward these two kinds of cancer cells. For example the nanosystem at 20 nm induced 58.6% HepG2 cell apoptosis, which was much higher than that of other two Ru@MSNs at 40(37.7%) and 80 nm (34.4%). These results further indicated that the different sized Ru@MSNs nanosystems efficiently induced hepatocellular carcinoma cells apoptosis, and exhibited the size-dependent effects toward cancer cells.

### 3.7. Overproduction of ROS stimulated by Ru@MSNs

ROS is a critical chemical signal activated by various anticancer chemotherapy drugs, and the excess ROS induces cancer cell death due to ROS mediated oxidative damage to biological molecules (Li et al., 2016). Excess ROS mainly stimulates DNA damage and protein modification, inducing cell apoptosis through downstream signaling pathways (Chang et al., 2017). Therefore, we examined ROS levels in HepG2 cells by DHE fluorescence assays.

As shown in Figure 3(b), ROS generation rapidly reached to maximum value after 5 min and then declines slowly in HepG2 cells. Under the same concentration, the different sized Ru@MSNs nanosystems stimulated more ROS overproduction than free RuPOP in cancer cells. While the different sized Ru@MSNs triggered ROS production in HepG2 cells was different. For example Ru@MSNs at 20 nm triggered the highest ROS levels in HepG2 cells was about 266.1%, and the other two particles sized Ru@MSNs at 40 and 80 nm triggered 147.1 and 177.2% of ROS overproduction in HepG-2 cells, which was much higher than that of free RuPOP (triggered about 110.5% ROS overproduction). Therefore, these results suggested that the different sized Ru@MSNs nanosystems induced ROS overproduction in HepG2 cells was much higher than that of free RuPOP, and suggested that the excess ROS was an important factor-induced size-dependent effects toward anticancer activity.

### 3.8. Activation of p53 signaling pathway by Ru@MSNs

p53 was known as a negative regulatory factor in the cell proliferation. Regulation of the p53 pathway has been identified as a significant factor in cell cycle regulation, DNA repair, cell differentiation, and apoptosis (Karimian et al., 2016; Kiraz et al., 2016). Therefore, we examined the protein level of phosphorylated p53 by immuno-fluorescence staining and western blotting. As shown in Figure 3(c), the expression of phosphorylated p53 in HepG2 cells was increased after treated with all the different sized Ru@MSNs comparing with free RuPOP, confirming that the nanosystems could promote p53 phosphorylation. To further illustrate the downstream signaling pathways regulated by p53 phosphorylation, we measured protein levels of P21 and DNA damage biochemical marker P-H_2_A.X by western blotting. As shown in Figure 3(d), different sized Ru@MSNs stimulated total p53 and phosphorylation elevation at Ser 9 site, and cell cycle regulation pathways p21 and p27 were also increased, regulated by phosphorylated p53. Up-regulated expression of DNA damage marker P-H_2_A.X showed that ROS attacked DNA, and then caused accumulation of phosphorylated p53. Therefore, ROS stimulated p53 phosphorylation plays a vital role in cell apoptosis triggered by Ru@MSNs.

### 3.9. *In vivo* antitumor activity and toxicity of Ru@MSNs

Biodistribution of different-sized Ru@MSNs in HepG2 xenografts nude mice was monitored with the animal fluorescence imaging technique at different time points. As shown in Figure 3(e), Ru@MSNs mainly accumulated in the tumor after intravenous injection for 72 h. We also investigated the different-sized Ru@MSNs and free RuPOP accumulated in the main organs, including the brain, heart, liver, spleen, lungs, kidney, and tumor. As shown in Figure 3(f), Ru@MSNs and free RuPOP accumulating in tumor were much higher than that in other organs after injection for 72 h. Besides, the smaller sized Ru@MSNs at 20 nm accumulating in tumor side was higher than the Ru@MSNs at 40 and 80 nm, but all the three kinds of nanosystem were much higher than the free RuPOP, indicating that the smaller sized Ru@MSNs was easier to penetrate in tumors.

**Figure 4.**
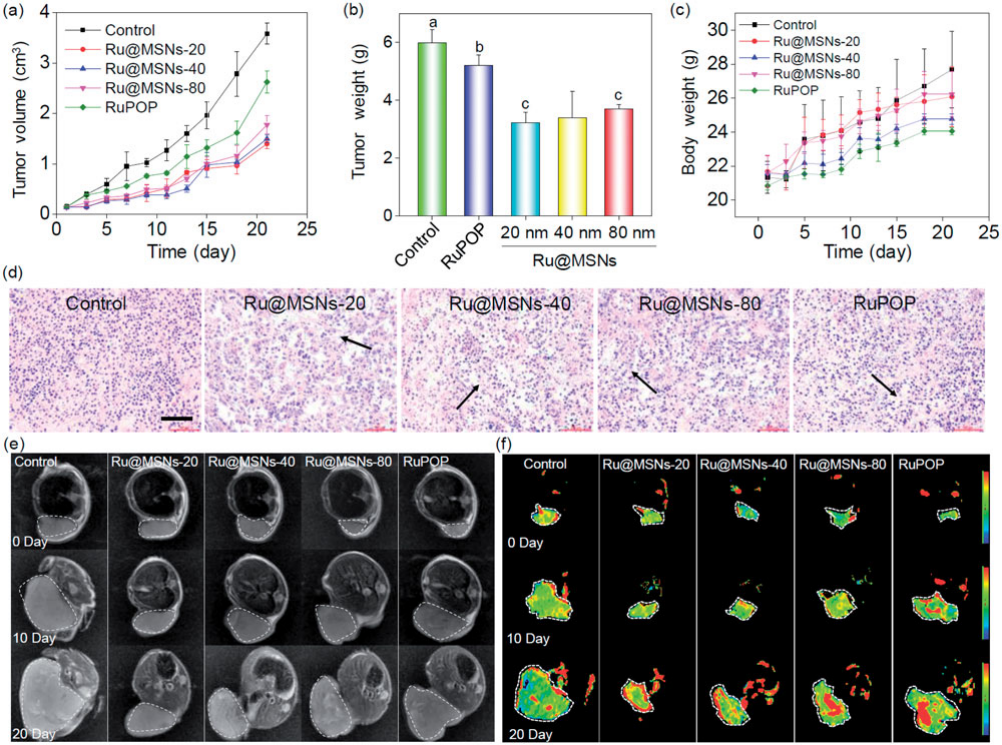
*In vivo* therapeutic effects of different-sized Ru@MSNs in HepG2 tumor-bearing mice. Changes in the tumor volume (a), tumor weight (b), and body weight (c) after treating with different-sized Ru@MSNs and free RuPOP at 0.2 mg/kg for 21 d. (d) H&E staining in tumor xenografts after treated with different-sized Ru@MSNs and free RuPOP. The scale bar is 100 μm. T2 weighted MRI (e) and pseudo color slow ADC (f) of HepG-2 tumor tissues after 10 and 21 d-treatment with different-sized Ru@MSNs and free RuPOP at 0.2 mg/kg. The tumor sites are in the back region and circled by dashed lines. Value represents means ± SD (*n* = 3).

We then examined the *in vivo* therapeutic effects of the different sized Ru@MSNs and free RuPOP toward HepG2 xenografts nude mice. As shown in Figure 4(a,b), the free RuPOP treatment did not significantly inhibit tumor growth, whereas the different sized Ru@MSNs nanosystems effectively inhibited tumor growth in the therapeutic efficacy of 20 nm.

Ru@MSNs was a little better than that of 40 and 80 nm nanosystems. For example the tumor growth inhibition of the Ru@MSNs at 20, 40, and 80 nm was about 46.16, 43.24, and 38.31%, while the inhibition of free RuPOP only reached to 12.97%. Body weights did not fluctuate significantly during all the different treatments (Figure 4(c)), suggesting that these treatments were well tolerated, and caused no acute side effects during therapy. We then detected the quantity of RuPOP in the main organs by ICP-MS, and found that RuPOP accumulating in tumor side was much higher than in other organs (Figure S8), suggesting the higher targeting of Ru@MSNs toward tumor *in vivo*.

**Figure 5.**
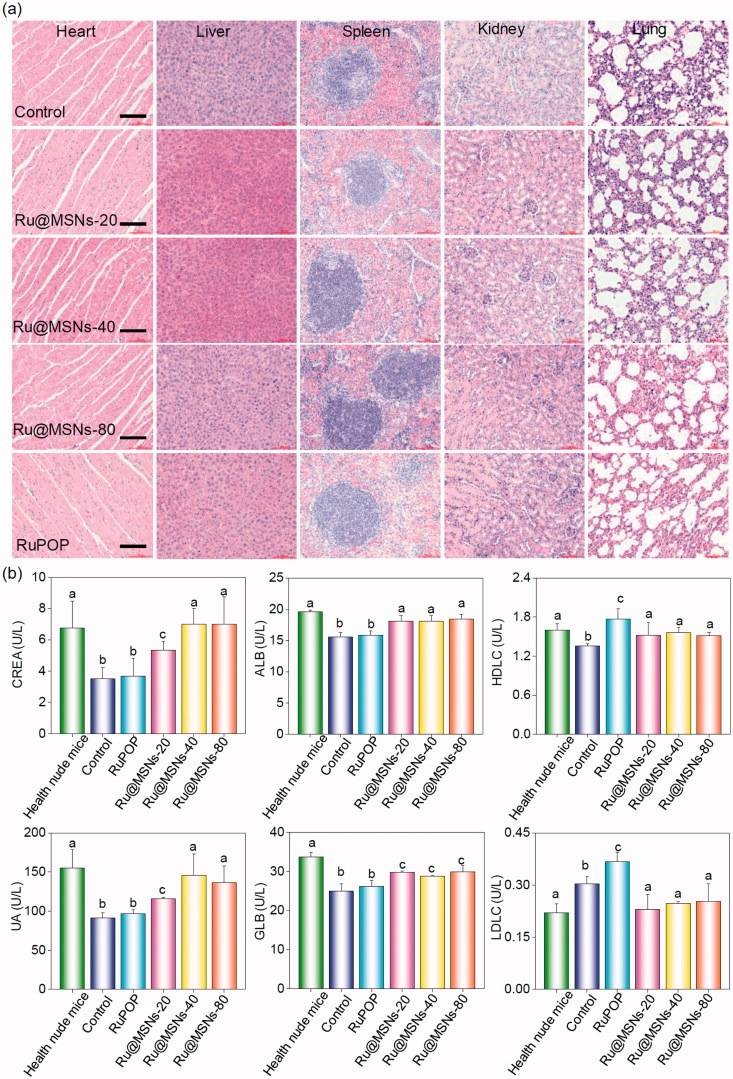
(a) The toxicity of the different-sized Ru@MSNs and RuPOP at 0.2 mg/kg on major organs after 21 d treatment. The scale bar is 100 μm. (b) Hematological analysis of healthy and tumor-bearing nude mice, and the treatment group of different-sized Ru@MSNs and RuPOP for 21 d. Value represents means ± SD (*n* = 3).

Furthermore, we also examined the histological changes of tumors tissues by H&E staining, and found that the tumors tissues exhibited different degrees of necrosis after treated with Ru@MSNs nanosystems and the free RuPOP (Figure 4(d)). *T*_2-_weighted magnetic resonance imaging (MRI) is more sensitive to examine tissue lesions and catch the signal of tissue edema or bleeding under emergency circumstances in living organisms during treatment. Therefore, we continuously monitored the changes of tumor status by the values of *T*_2-_weighted, and slow ADC in living tumor tissues after treated with Ru@MSNs and free RuPOP. As shown in Figure 4(e), *T*_2-_weighted images showed that the treatment groups of tumors were much smaller than the control. Especially the group of Ru@MSNs-20 nm, the tumor growth was obviously inhibited with the increase of treated time. Besides, the larger slow ADC signals (red) were observed in the tumor site after treated by different sized Ru@MSNs and RuPOP for 20 d, indicating low density of cancer cell and higher degree of tumor cell necrosis inside the tumors after treatment (Figure 4(f) and S9). For example the slow ADC values of the control group decreased from 0.412 (0 d) to 0.276 (20 d), while after treated with Ru@MSNs and RuPOP, the slow ADC values of all the treated tumors was increased, especially the Ru@MSNs at 20 nm which increased from 0.383 (0 d) to 0.627 (20 d). These results further indicated that the different-sized Ru@MSNs nanosystems effectively inhibited tumor growth, especially the Ru@MSNs at 20 nm. Furthermore, we also examined the histological changes in the major organs of the treatment group mice by H&E staining to evaluate the security of Ru@MSNs nanosystems. As shown in Figure 5(a), no significant impairment or inflammation was found in the major organs under the experimental conditions, including heart, liver, spleen, lung, and kidney. Then, we also conducted the hematological analysis to examine the effects of different sized Ru@MSNs nanosystems on the functions of kidney and liver of nude mice. As shown in Figure 5(b), the different sized Ru@MSNs significantly alleviated the damages of the functions of kidney and liver by tumor formation, as reflected by reversion in these blood biochemical values to the level of healthy group. These results further confirm the safety and application potential of Ru@MSNs nanosystems as anticancer agents.

### 3.10. Anti-drug resistance of Ru@MSNs through suppressing drug efflux

**Figure 6.**
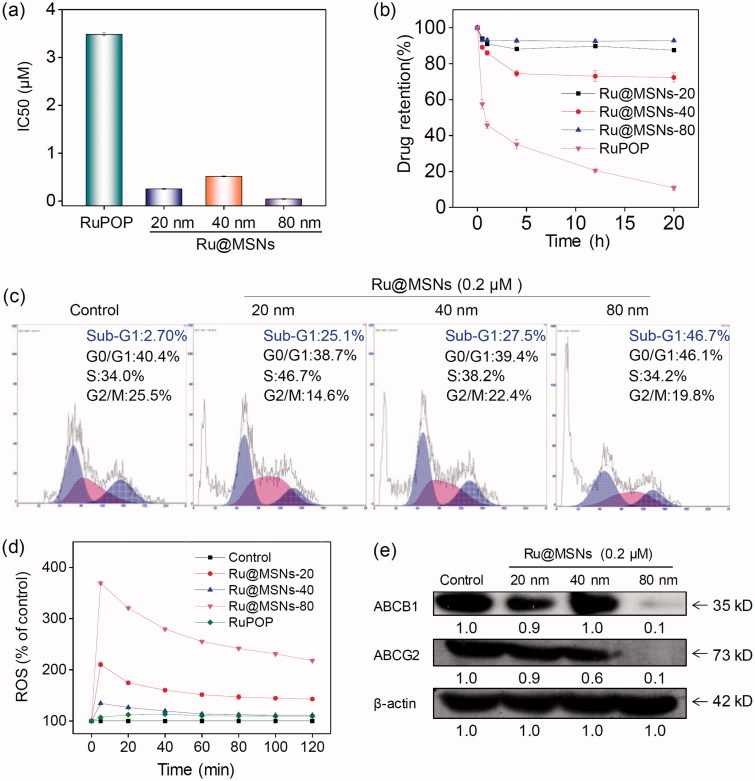
Anti-drug resistance of different-sized Ru@MSNs nanosystems through suppressing drug efflux. (a) IC_50_ for the RuPOP and different-sized Ru@MSNs toward the drug resistance cells of R-HepG2. (b) Quantitative analysis of drug retention for the different-sized Ru@MSNs in R-HepG2 cells. (c) Flow cytometric analysis of R-HepG2 cells exposed with 0.2 μM of different-sized Ru@MSNs for 24 h. (d) ROS overproduction in R-HepG2 cells exposed to RuPOP and Ru@MSNs. (e) Inhibition of ABC family by Ru@MSNs. Value represents means ± SD (*n* = 3).

MDR is one of the most essential approaches to drug efflux from cells which reduces the effective dose at cancer treatment, thus become the main obstacle in clinical cancer therapy. Therefore, overcoming MDR has been an important focus for improving cancer chemotherapy efficacy and preventing tumor recurrence. We then examined the anticancer activity toward DOX-resistance R-HepG2 cells by MTT assay. As shown in Figure 6(a), all the three Ru@MSNs nanosystems at 20, 40, and 80 nm showed much higher cytotoxicity toward R-HepG2 cells than the free RuPOP. For example the IC50 value of Ru@MSNs at 80 nm was lowest, which was about 0.044 μM. And the IC50 value of other two particles sized Ru@MSNs at 20 and 40 nm were about 0.253 and 0.515 μM, respectively, which was lower than the free RuPOP (3.482 μM). Drug efflux in tumors was an important factor leading to inefficient treatment in cancer chemotherapy. We then examined the intracellular retention efficacy of Ru@MSNs and free RuPOP in R-HepG2 cells. As shown in Figure 6(b), we found that the intracellular RuPOP in R-HepG2 cells decreased significantly with time increased, while all the three particle sizes of Ru@MSNs nanosystems could remain for a longer time in cells. For instance, after 20 h, the retention efficacy of free RuPOP in R-HepG2 cells was only 11.2%, while all the three kinds of Ru@MSNs at 20, 40, and 80 nm remain for a higher retention efficacy in R-HepG2 cells, which was about 86.7, 71.6, and 92.7%, respectively. These results indicated that Ru@MSNs could accumulate in R-HepG2 cells and remain for a long time compared with the free RuPOP, thus revealing the higher anti-drug resistance toward R-HepG2 cells. We also detected R-HepG2 cell cycles after treated with Ru@MSNs by flow cytometry. As shown in Figure 6(c), the lager sized Ru@MSNs at 80 nm exhibited much higher efficiency to induce cell apoptosis than the Ru@MSNs at 40 and 20 nm. For instance, Ru@MSNs at 80 nm induced 46.7% cell apoptosis, but Ru@MSNs at 40 and 20 nm only induced 27.5 and 25.1% cell apoptosis. These results indicated that Ru@MSNs inhibited the proliferation of DOX-resistance R-HepG2 cells through induced cell apoptosis. We also found that the different sized Ru@MSNs and the free RuPOP triggered ROS production in R-HepG2 cells was different. As shown in Figure 6(d), for example ROS levels treated with free RuPOP was about 107.0% at 5 min, which was lower than the Ru@MSNs treated cells. Besides, the highest ROS overproduction in R-HepG2 cells was triggered by the larger sized Ru@MSNs at 80 nm, which was reached to 369.3%. These results suggested that the different sized Ru@MSNs nanosystems achieved higher anti-drug resistance through inducing ROS overproduction in R-HepG2 cells.

ABC family proteins have been identified as regulatory factors for drug absorption, metabolism, and efflux, and are essential for MDR, particularly for the drug efflux pump P-glycoprotein encoded by the *ABCB1* gene (Locher, 2016; Song et al., 2016). Inhibiting ABC family protein expression is a direct and efficient approach to overcome MDR that avoids drug efflux and sustains adequate intracellular drug dosage. Therefore, we examined the ABC family protein expression levels in R-HepG2 cells after treated with different sized Ru@MSNs nanosystems. As shown in Figure 6(e), the expression levels of two key proteins, ABCB1 and ABCG2, were suppressed by all the different sized Ru@MSNs nanosystems, and the larger Ru@MSNs at 80 nm exhibited more effectively inhibition than the other two particles sized Ru@MSNs. Therefore, the different sized Ru@MSNs could overcome MDR by inhibiting ABCB1 and ABCG2, and consequently enhancing DNA damage mediated cancer cell apoptosis.

## 4. Discussion

Nanotechnology-based delivery systems display potent applications in cancer therapy, owing to the EPR effect of nanoparticles (Talelli et al., 2015; Yu & Zheng, 2015). Nanoparticles with EPR effect permeate into tumor sites from its leaky epithelium and discontinuous microvasculatures, which formed by the rapid growth of tumors (Peer et al., 2007). Recent studies have supported that particle size, morphology, and surface property are the important factors impact their EPR effect (Matsumura & Maeda, 1986). Various of nanoparticles, such as selenium nanoparticle, liposome, gold nanoparticle, and polymers, are used to enhanced cancer therapy. Among various nanoparticle, MSN is regarded as excellent drug delivery system in cancer therapy because of high drug loading capability, high biocompatibility, low toxicity, and easy for surface-modification (Hu et al., 2015; Chen & Shi, 2016), which show great potential in cancer treatment. For instance, Shi et al. reported various of MSNs nanosystems, like TAT-peptide modified MSNs loading DOX could overcome drug resistance via nuclear target (Pan et al., 2012). Zink et al. synthesized a MSNs co-delivery system of DOX and siRNA to anti-breast cancer achieving combine treatment (Meng et al., 2013). According to our previous reported, He et al. synthesized a MSNs as delivery nanosystem as delivery of gold (III) complex to enhance anticancer activity without toxicity (He et al., 2014a), and Mo et al. found that 40 nm MSNs loading DOX exhibit remarkable capacity to overcome blood-brain barrier (Mo et al., 2016). However, the size effects of MSNs nanosystems on cancer treatment and overcome MDR do need to further clarify.

This study used reactant and ratio to control MSN particle size, and synthesized three nanosystems with different particle size at about 20, 40, and 80 nm. These nanodrug delivery systems are expected to realize targeted recognition between cancer and normal tissue, promoting anticancer therapeutic efficiency and overcoming MDR in cancer therapy. The different sized MSNs were conjugated with FA to facilitate selectivity toward hepatocellular carcinoma cells, and loaded with ruthenium complex, which possessed excellent anticancer activity. Hence, the functionalized nano-delivery systems specifically identified and bound to the HepG2 cells, with overexpressed FR, consequently promoting cellular uptake and retention toward HepG2 and DOX-resistant R-HepG2 cells. The Ru@MSNs could be uptaken by cancer cells promptly, achieving intracellular drug release and providing higher effective dosages, and exhibited higher stability and hematological security in body blood circulation. Furthermore, the particle size of Ru@MSNs significantly affects anticancer activity toward different cancer cells. The smaller size (20 nm) of nanodrugs exhibit higher anticancer activity toward HepG2 cells, while the larger size (80 nm) of Ru@MSNs exhibit higher inhibitory effect toward DOX-resistant R-HepG2 cells, which was related to the drug uptake and retention in cancer cells. The Ru@MSNs nano-delivery systems displayed superior selectivity and anticancer activity compared with free RuPOP, and stimulated ROS overproduction that induced hepatocellular carcinoma cells apoptosis. More significantly, the larger size (80 nm) of Ru@MSNs showed superior potential for overcoming MDR toward resistant R-HepG2 cells, due to suppressing ABC family drug efflux pump expression. More importantly, the different sized Ru@MSNs nanosystems exhibited high *in vivo* antitumor activity, especially the nanosystems at 20 nm, and all the nanosystems exhibited low toxicity *in vivo*. These preliminary results confirm that MSN nanodrug delivery system act as potential anticancer approach and exhibit size-dependent effects toward anticancer activity and suppressing cancer MDR.

## Supplementary Material

IDRD_Chen_et_al_Supplemental_Content.doc
