# Correction to: Ma et al., Controlled synthesis and size effects of multifunctional mesoporous silica nanosystem for precise cancer therapy

**DOI:** 10.1080/10717544.2018.1439352

**Published:** 2018-02-12

Ma B, He L, You Y, Mo J, and Chen T. (2018). Controlled synthesis and size effects of multifunctional mesoporous silica nanosystem for precise cancer therapy. *Drug Delivery*. https://doi.org/10.1080/10717544.2018.1425779.

When the above article was first published online, the affiliation for the authors was wrong.

This has now been corrected in the online version.

