# Supplementary material for: Controlled synthesis and size effects of multifunctional mesoporous silica nanosystem for precise cancer therapy
Source: Drug Deliv. 2018 Jan 15;25(1):293–306. doi: 10.1080/10717544.2018.1425779 (PMC6058695; doi:10.1080/10717544.2018.1425779)
Supplement: IDRD_Chen_et_al_Supplemental_Content.doc [file IDRD_A_1425779_SM4546.doc]

**Electronic Supporting Information (ESI)**

**Controlled synthesis and size effects of multifunctional mesoporous silica nanosystem for precise cancer therapy**

Bin Ma, Lizhen He*, Yuanyuan You, Jianbin Mo, Tianfeng Chen*

*Department of Chemistry, Jinan University, Guangzhou 510632, China.*

****Corresponding author***: Tel: +86 20885225962.

**E-mail**: tchentf@jnu.edu.cn; hlz6371@jnu.edu.cn.

**RESULTS**

**Figure S1**. Stability of the different-sized MSNs at 20 nm, 40 nm and 80 nm. Value represents means ± SD (n=3).

**Figure S2** Stability of the different-sized MSNs at 20 nm, 40 nm and 80 nm in Fetal bovine serum (a) and DMEM contain 10% FBS (b). Value represents means ± SD (n=3).

**Figure S3**. FTIR spectra of PEI and PEI-FA (PEI conjunct with FA).

**Figure S4** The cells viability after treated with different-sized MSNs carriers, PEI-FA and MSNs-PEI-FA. Value represents means ± SD (n=3).

**Figure S5**. The expression of FA receptor in L02, HepG2 and R-HepG2 and cells.

**Figure S6.** Cellular uptake of different-sized Ru@MSNs in HepG2 (a) and L02 (b) cells after blocked with 1 mg/ml FA for 2 h; Cells were treated with 1 *μ*M of Ru@MSNs and free RuPOP for 4 h and 8 h. (c) Quantitative analysis of drug retention for the different-sized Ru@MSNs in HepG2 cells. Value represents means ± SD (n=3).

**Figure S7**. Hemolysis ratio of the different-sized MSNs nanosystems and the free RuPOP. Value represents means ± SD (n=3).

**Figure S8.** Quantitative analysis of the RuPOP concentration in main organs in 21 d-treatment with Ru@MSNs and free RuPOP. Value represents means ± SD (n=3).

**Figure S9**. Slow ADC of IVIM-DWI parameters of tumor tissue in different treatment group by MRI analysis. Value represents means ± SD (n=3).
